# Protective Effects of Antioxidant Mix Pre-Treatment Against Visible Light-Induced Damage in Dark Skin Phototype

**DOI:** 10.3390/antiox15091072

**Published:** 2026-08-26

**Authors:** Anna Guiotto, Malak Alghamdi, Robyn Hickerson, Michael Conneely, Hina Choudhary, Patricia Brieva, Yunsook Lim, Alessandra Pecorelli, Giuseppe Valacchi

**Affiliations:** 1 Plants for Human Health Institute, NC Research Campus, NC State University, Raleigh, NC 27695, USA; aguiott@ncsu.edu (A.G.); malgham@ncsu.edu (M.A.); 2 Ten Bio Technologies Inc., NC Research Campus, Kannapolis, NC 28081, USA; 3 SkinCeuticals, New York, NY 10021, USA; 4 Department of Food and Nutrition, Kyung Hee University, Seoul 02447, Republic of Korea; 5 Plants for Human Health Institute, Department of Food, Bioprocessing and Nutrition Sciences, NC Research Campus, NC State University, Raleigh, NC 27695, USA; 6 Department of Environment and Prevention Sciences, University of Ferrara, 44121 Ferrara, Italy

**Keywords:** premature aging, antioxidant mixture, oxidative stress, hyperpigmentation, photoaging

## Abstract

Visible light (VL) accounts for approximately 50% of the solar radiation reaching Earth’s surface and has emerged as a major contributor to skin photoaging and pigmentation disorders, particularly in individuals with darker Fitzpatrick skin phototypes. Increasing evidence suggests that VL exposure induces oxidative stress, inflammation, and melanogenesis by generating reactive oxygen species. In this study, we investigated the protective effects of a topical antioxidant formulation (AOX Mix) containing 15% ascorbic acid, 0.5% ferulic acid, and 1% tocopherol against VL exposure in ex vivo skin biopsies from donors with Fitzpatrick skin phototypes IV–V. To preserve physiological tissue tension and closely replicate in vivo skin responses, human skin explants were maintained using the TenSkin™ culture system. Samples were pretreated with AOX Mix for 30 min, then exposed to VL for 8 h, and collected on Days 2 and 7 for histological and molecular analyses. Our results demonstrated that prolonged exposure to VL disrupted redox homeostasis and induced structural alterations in skin explants, accompanied by increased markers of oxidative stress and pigmentation. In contrast, pre-treatment with AOX Mix significantly attenuated these effects, preserving tissue architecture and reducing molecular indicators of photodamage. In addition, we observed the co-localization of 4-hydroxynonenal and collagen type I, suggesting that oxidative post-translational modification of collagen may contribute to its loss. These findings suggest that antioxidant-based interventions, such as AOX Mix, may be a promising strategy for protecting dark skin against VL exposure.

## 1. Introduction

Visible light (VL), with wavelengths ranging from 400 to 700 nm, accounts for approximately 50% of the solar radiation reaching the Earth’s surface. In addition to natural sunlight, VL is also emitted by various artificial sources, including lasers, LED lamps, and fluorescent lighting [1]. Although ultraviolet (UV) radiation has historically been regarded as the principal driver of photoaging and pigmentation disorders [2,3,4,5], accumulating evidence suggests that VL also triggers significant cutaneous responses, with blue light in particular contributing to oxidative stress, inflammation, and dysregulation of melanogenesis [6]. Notably, approximately 4–7% of incident VL is reflected from the skin surface, largely independent of wavelength and skin phototype [7]. For example, Kollias and Barger [8] demonstrated that irradiation of the lower inner arm with 390–1700 nm light induced skin pigmentation that remained visible for up to eight weeks. However, cutaneous responses to VL exposure vary by skin phototype. Repeated low-dose VL irradiation has been shown to induce persistent pigmentation, especially in individuals with darker skin phototypes (Fitzpatrick IV–VI) compared with those with lighter skin phototypes [9]. Compared with lighter skin types, darker skin contains larger, more melanized melanosomes, which, in addition to providing enhanced natural protection, may also increase the risk of post-inflammatory hyperpigmentation and uneven skin tone [10].

Accumulating evidence suggests that the biological effects of VL exposure are mediated, at least in part, by the generation of reactive oxygen species (ROS). Exposure of human skin equivalents to VL has been reported to promote ROS production, trigger pro-inflammatory cytokine release, enhance matrix metalloproteinase expression, and activate oxidative stress-responsive signaling pathways [11,12]. Therefore, antioxidant-based strategies have emerged as promising approaches to mitigate visible light-induced skin damage and pigmentation. In particular, formulations containing combinations of bioactive compounds may provide broad-spectrum protection by attenuating oxidative stress, reducing DNA damage, and preserving extracellular matrix homeostasis. In the present study, we investigated the protective effects of a topical antioxidant cosmeceutical mixture (AOX Mix) containing 15% ascorbic acid, 0.5% ferulic acid, and 1% tocopherol (CE Ferulic, SkinCeuticals Inc., New York, NY, USA) against VL exposure in skin biopsies obtained from patients with dark skin phototypes (Fitzpatrick IV–V). In addition, to mimic the physiological skin tension, the TenSkin™ model was used (Ten Bio Technologies, Inc., NC, USA, https://ten-bio.com (accessed on 17 December 2025)). This model provides a reliable approach for mimicking the in vivo environment by maintaining tissues under physiological tension and reproducing physiologically relevant responses to external stimuli [13,14,15,16]. In this study, skin explants were pretreated with AOX Mix for 30 min and subsequently exposed to VL radiation for 8 h. Biopsies were collected on days 2 and 7. Then, morphological changes, melanin production, and several markers related to oxidative stress, DNA damage, and skin structure were assessed. Our results demonstrate that VL exposure impairs redox homeostasis and induces structural alterations in the skin of individuals with dark skin phototypes. Notably, the co-localization of Collagen 1 and 4-hydroxynonenal (4HNE) was demonstrated for the first time, suggesting a previously unrecognized mechanism of redox-modulated f collagen modification. Importantly, AOX Mix mitigated these changes, demonstrating a protective effect against photodamage and supporting its potential use as a photoprotective intervention.

## 2. Materials and Methods

### 2.1. Culture and Exposure of Ex Vivo Human Skin Explants

Ex vivo human skin explants from three healthy donors with dark skin phototypes IV–V undergoing elective abdominoplasty were obtained from Ten Bio and cultured under physiologically relevant tension (TenSkin™ TS-18, Ten Bio Technologies, Inc., Kannapolis, NC, USA) (IRB 21-TEN-101). Compared with conventional cell culture systems and animal models, ex vivo skin explants preserve the native tissue architecture and cellular heterogeneity of the human skin [17,18,19]. The TenSkin™ ex vivo model further maintains physiological tissue tension while preserving key structural and functional components, including the extracellular matrix, epidermal barrier, hair follicles, sweat glands, and resident immune cells. By maintaining cell–cell and cell–matrix interactions, this model more closely reproduces the in vivo skin microenvironment, providing a physiologically relevant platform for evaluating tissue responses to environmental stressors [13,16]. Skin models were transferred to untreated culture plates containing standard culture medium provided by the company, using sterile technique, and incubated in a humidified atmosphere of 95% air and 5% CO_2_ at 37 °C overnight to allow tissue recovery [20,21]. Before VL exposure, skin biopsies were pretreated with AOX Mix for 30 min and then returned to the incubator. After treatment, excess serum was gently removed with sterile gauze, and models were exposed to VL for 8 h/day in a dedicated incubator equipped with a VL irradiation system that maintained a constant temperature throughout the exposure period. The irradiance at the sample surface was 0.011 W/cm^2^, as measured using an MT-912 light meter (Supplementary Figure S1). Control models not subjected to VL exposure were maintained in a separate incubator at 37 °C in a humidified atmosphere of 5% CO_2_ and 95% air. At the end of each exposure period, the culture medium was replaced, and the models were returned to the incubator until the next exposure cycle. Biopsies were harvested on days 2 and 7 for downstream analyses.

### 2.2. Hematoxylin and Eosin (H&E) Staining

Paraffin-embedded tissue samples were sectioned at 5 µm, mounted on slides, and deparaffinized in Histoclear 2 × 3 min. Subsequently, the sections were rehydrated through a graded series of ethanol solutions of decreasing concentration, ending in water. Nuclei were stained with Mayer hematoxylin for 5 min, then rinsed under running tap water for 5 min. Sections were then blued by immersion in Scott’s tap water for 2 min. Slides were briefly rinsed in distilled water and then immersed in 70% ethanol for 3 min. Cytoplasmic staining was performed using eosin for 1 min, followed by a brief rinse in 95% ethanol. Slides were subsequently dehydrated through graded ethanol solutions of increasing concentration, followed by Histoclear. Finally, slides were mounted using a DPX mounting medium and allowed to dry before imaging. Images were acquired using a Leica DM2000 LED microscope (Leica Microsystems GmbH, Wetzlar, Germany) equipped with ManualWSI software 2021 Edition (Microvisioneer, Esslingen am Neckar, Germany).

### 2.3. Fontana–Masson

Fontana–Masson stain was performed using a commercial kit (Abcam, Waltham, MA, USA; cat. no. ab150669) according to the manufacturer’s protocol. Briefly, 5 µm tissue-thick sections were deparaffinized in Histoclear, rehydrated through graded alcohols of decreasing concentration, and incubated in 10% ammoniacal silver nitrate solution at 60 °C for 45 min in the dark. After three washes with distilled water, the slides were incubated in gold chloride solution for 30 s and washed three times with distilled water. The slides were then incubated in sodium thiosulfate solution for 2 min at room temperature (RT). Following three additional washes with distilled water, the sections were counterstained with nuclear fast red for 5 min and rinsed again. The samples were then dehydrated through graded alcohols of increasing concentration, cleared in Histoclear, and mounted using DPX mounting medium (Watkins & Doncaster, Herefordshire, UK). Images were acquired using a Leica DM2000 LED microscope (Leica Microsystems GmbH, Wetzlar, Germany) equipped with ManualWSI software 2021 Edition (Microvisioneer, Esslingen am Neckar, Germany).

### 2.4. Immunohistochemistry

For immunohistochemistry analysis, paraffin sections of skin explants (4 µm) were deparaffinized in xylene and rehydrated through graded alcohol. The sections were then subjected to antigen retrieval by immersing the slides in a plastic jar containing a 10 mM sodium citrate buffer (AP-9003500, ThermoFisher Scientific, Waltham, MA, USA) (pH 6.0) and heating them in a water bath at 96 °C for 8 min. After cooling the slides for 30 min at RT, the tissues were washed twice in PBS for 5 min each and blocked in 2% BSA in PBS for 45 min at RT. Sections were then incubated overnight at 4 °C with primary antibodies diluted in PBS-BSA 0.25% at different dilutions based on the specific antibody: 4HNE (cat. AB5605, Merk Millipore, Darmstadt, Germany, 1:500); Collagen 1 (cat. ab138492, Abcam Waltham, MA, USA, 1:2000); Matrix Metalloproteinase-2 (MMP2) (cat. 436000, ThermoFisher Scientific, Waltham, MA, USA, 1:250); Tissue Inhibitor of Metalloproteinase 1 (TIMP1) (cat. MA1-773, ThermoFisher Scientific, Waltham, MA, USA, 1:250); and Phosphorylated Histone H2A.X (PH2AX) (cat. sc-517348, Santa Cruz biotechnology, Dallas, TX, USA, 1:50). The following day, sections were washed 3 times in PBS for 5 min and then incubated with fluorochrome-conjugated secondary antibodies (A11004 Alexa Fluor 568, A11008 Alexa Fluor 488 Invitrogen, Carlsbad, CA, USA; ThermoFisher Scientific, Waltham, MA, USA) at a dilution of 1:1000 in PBS-BSA 0.25% for 1 h at RT. Nuclei were then stained with DAPI (D1306, Invitrogen, Carlsbad, CA, USA; ThermoFisher Scientific, Waltham, MA, USA) for 1 min in PBS at RT, washed 3 times in PBS for 5 min, and mounted using PermaFluor mounting media (ThermoFisher Scientific, Waltham, MA, USA). Images were acquired using a Zeiss Z1 AxioObserver LSM10 confocal microscope (Carl Zeiss Microscopy GmbH, Jena, Germany) equipped with a 40× objective. Images were quantified using ImageJ (Version 1.54; National Institutes of Health, Bethesda, MD, USA) [22,23,24].

### 2.5. RNA Extraction and Quantitative Real-Time PCR (RT-PCR)

Total RNA was extracted from skin biopsies after mechanical disruption and homogenization (4 min at 30 Hz; using 5 mm metal beads in RLT buffer) using a TissueLyser (Qiagen, Germantown, MD, USA). Total RNA was isolated from the biopsies using an RNeasy Plus 96 Kit (Qiagen Inc., USA, #74192) following the manufacturer’s protocol and stored at −80 °C. cDNA was synthesized from approximately 100 ng of total RNA using the High-Capacity cDNA Reverse Transcription Kit (Applied Biosystems, Foster City, CA, USA, #4368813) following the manufacturer’s protocol and stored at −20 °C. RT-qPCR was performed using a QuantStudio 3 Real-Time PCR System (Applied Biosystems, USA) and TaqMan Real-Time qPCR Assays (Applied Biosystems, USA) to quantify relative mRNA expression using the ΔΔCt method. Ribosomal protein S13 (RPS13; TaqMan ID Hs01011487_g1) was used as the endogenous control for all qPCR experiments. The expression of HMOX1 was quantified using a pre-designed TaqMan Gene Expression Assay (Thermo Fisher Scientific, Waltham, MA, USA; Assay ID: Hs01110250_m1).

### 2.6. Statistics

Statistical analysis was performed using GraphPad Prism 11 (GraphPad Software, San Diego, CA, USA). A one-way ANOVA followed by Tukey’s multiple comparisons test was applied. A *p*-value < 0.05 was considered significant, and all data are reported as mean ± standard error of the mean (SEM).

## 3. Results

### 3.1. AOX Mix Prevents Hyperpigmentation in Dark Skin Explants After VL Exposure

**Figure 1:**
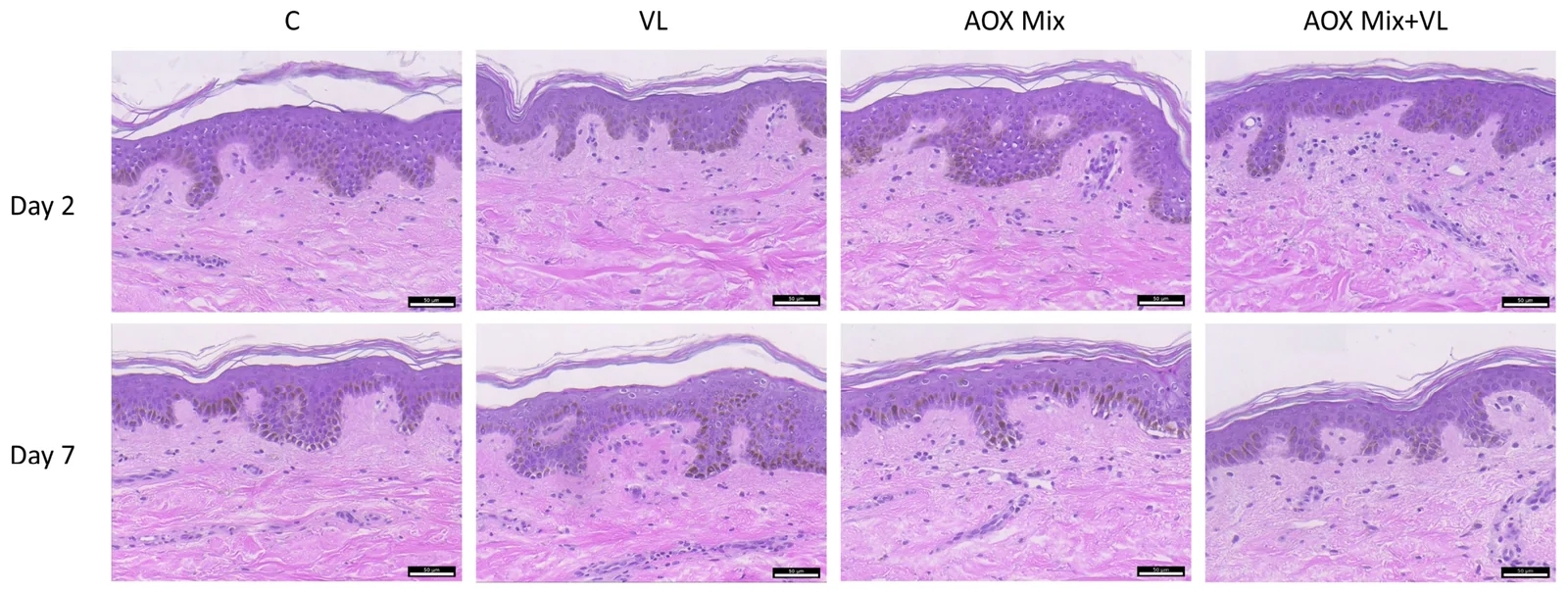
VL exposure does not alter tissue morphology. H&E staining of ex vivo human skin biopsies after pre-treatment with AOX Mix and exposure to VL for 2 or 7 days. Experimental groups: C, untreated control; VL, visible light exposure; AOX Mix, AOX Mix treatment alone; AOX Mix + VL, AOX Mix pre-treatment followed by visible light exposure. D2 and D7 indicate tissue collection on days 2 and 7, respectively. Original magnification ×20; scale bar 50 µm.

**Figure 2:**
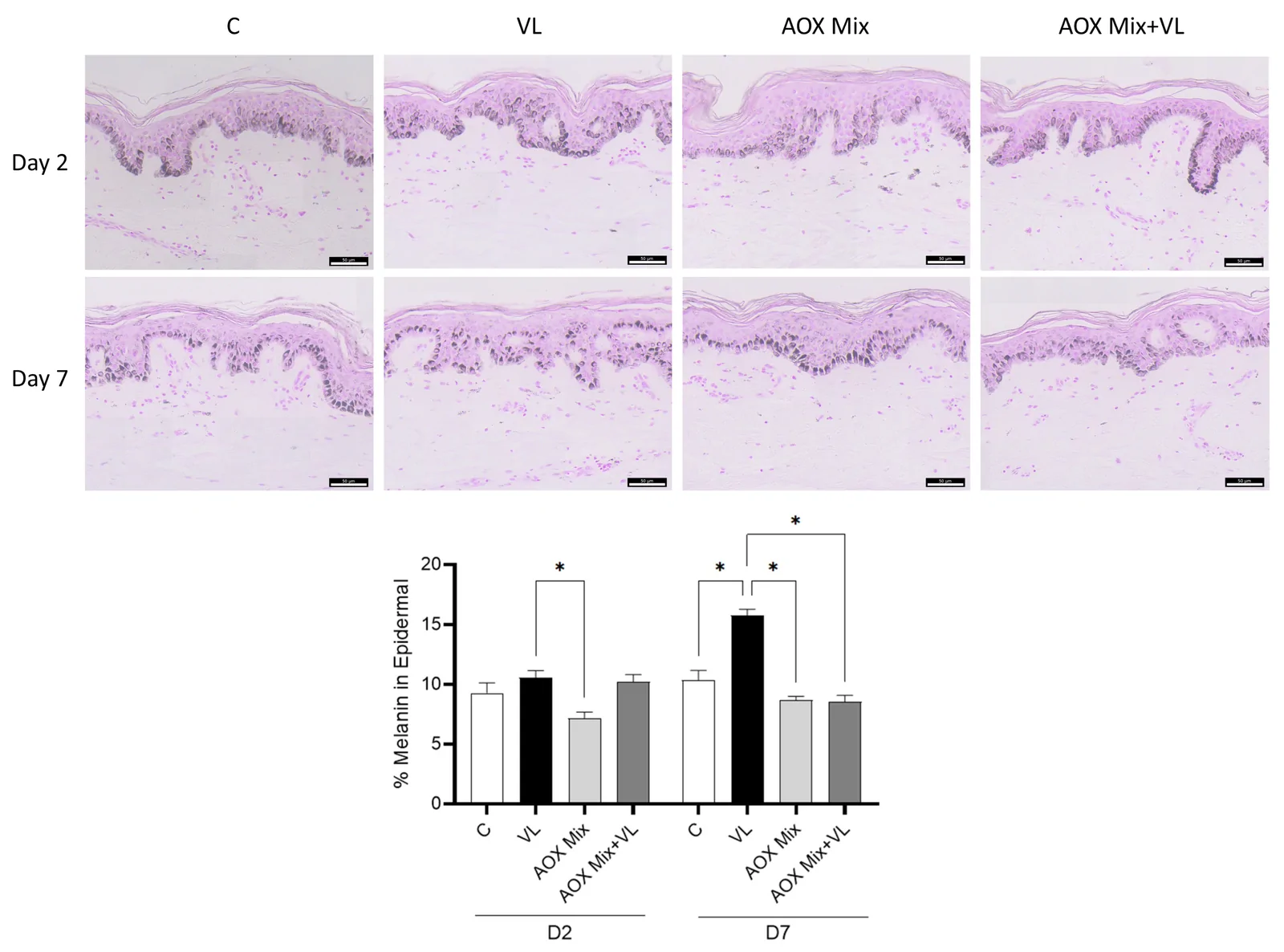
VL exposure increases melanin pigment in tissues exposed to VL. Fontana–Masson staining of ex vivo human skin biopsies after pre-treatment with AOX Mix and exposure to VL for 2 or 7 days. Experimental groups: C, untreated control; VL, visible light exposure; AOX Mix, AOX Mix treatment alone; AOX Mix + VL, AOX Mix pre-treatment followed by visible light exposure. D2 and D7 indicate tissue collection on days 2 and 7, respectively. Original magnification ×20; scale bar 50 µm. Significant differences are indicated with * *p*-value < 0.05. Data are expressed as mean ± SEM and analyzed using one-way ANOVA with Tukey’s multiple comparison post-hoc test.

Hematoxylin and eosin staining was initially performed to evaluate tissue integrity. The analysis showed that the VL exposure did not induce morphological changes in the TenSkin™ skin model at either D2or D7, indicating that the experimental conditions were well tolerated (Figure 1). Next, the potential protective effect of AOX Mix formulation against VL-induced hyperpigmentation in dark skin was evaluated using Fontana–Masson staining, which visualizes melanin deposition in skin tissue. As shown in Figure 2, VL exposure induced a marked increase in melanin deposition, particularly at day 7 (D7). Notably, pre-treatment with AOX Mix effectively reduced this increase in melanin accumulation following VL exposure at the later time point (D7).

### 3.2. AOX Mix Attenuates DNA Damage and Oxidative Stress Caused by VL Exposure

**Figure 3:**
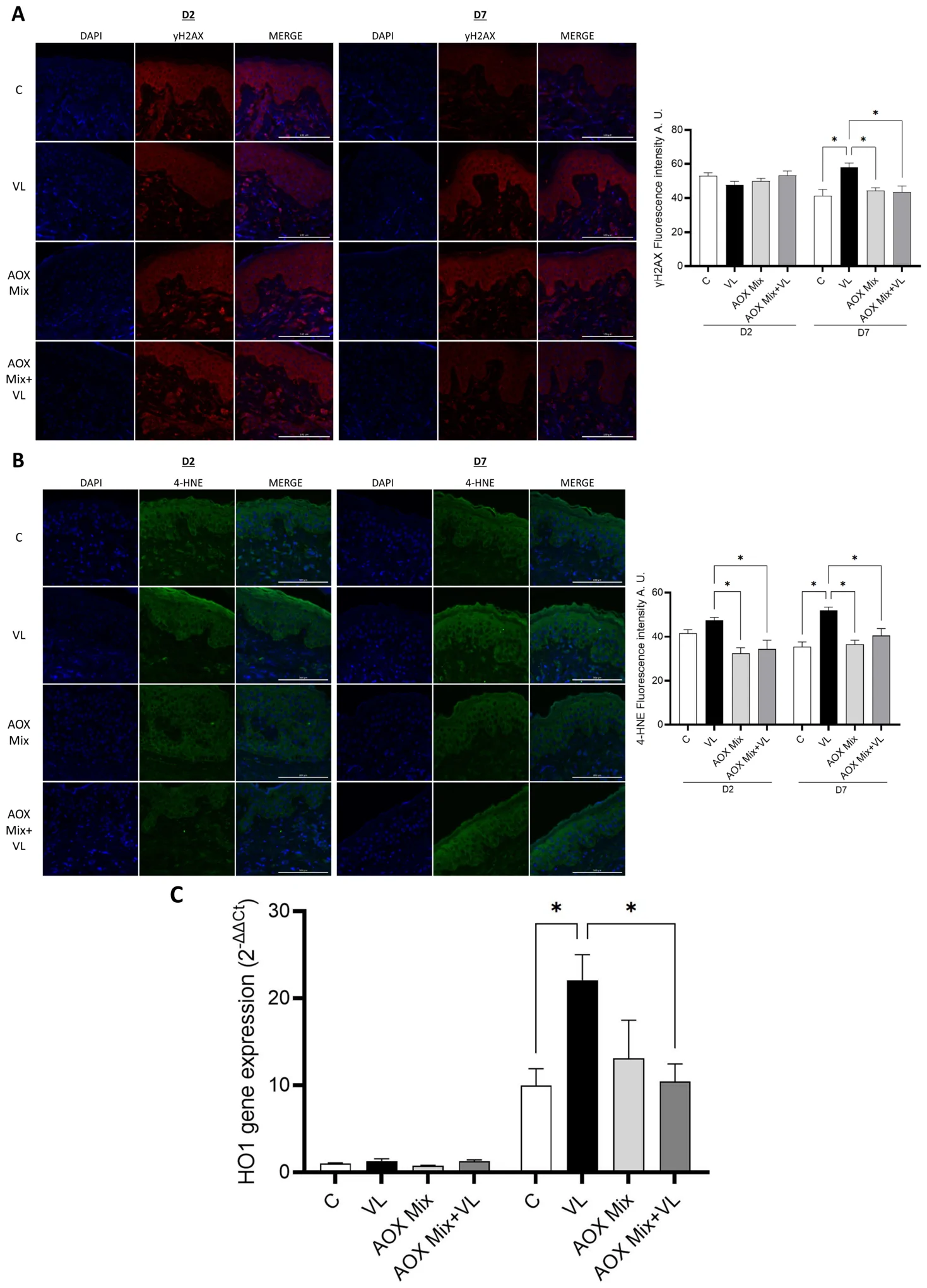
(**A**) Representative images of pH2AX protein expression in ex vivo human skin biopsies after topical pre-treatment with AOX Mix formulation and exposure to VL radiation for 2 or 7 days (pH2AX, red; DAPI, blue). The right panel shows signal quantification of the fluorescence intensity. (**B**) Representative images of 4HNE protein adduct expression in ex vivo human skin biopsies after topical pre-treatment with AOX Mix formulation and exposure to VL radiation for 2 or 7 days (4HNE, green; DAPI, blue). The right panel shows signal quantification of the fluorescence intensity. Images were captured at 40x magnification. (**C**) qRT-PCR analysis of HO-1 transcript expression. Experimental groups: C, untreated control; VL, visible light exposure; AOX Mix, AOX Mix treatment alone; AOX Mix + VL, AOX Mix pre-treatment followed by visible light exposure. D2 and D7 indicate tissue collection on days 2 and 7, respectively. Significant differences are indicated with * *p*-value < 0.05. Data are expressed as mean ± SEM and analyzed using one-way ANOVA with Tukey’s multiple comparison post-hoc test.

To assess and quantify DNA double-strand breaks induced by VL exposure, as well as the potential protective effect of AOX Mix pre-treatment, immunofluorescence staining for γH2AX, the phosphorylated form of histone H2AX at Ser139, which rapidly accumulates at sites of DNA damage [25], was evaluated. As shown in Figure 3A, VL exposure significantly increased γH2AX expression at day 7. Of note, AOX Mix pre-treatment effectively prevented this increase, maintaining γH2AX levels comparable to those observed in untreated control samples. In addition, to evaluate the ability of VL to induce oxidative damage, lipid peroxidation was assessed by immunofluorescence detection of 4-HNE protein adducts, a highly reactive unsaturated aldehyde generated during lipid peroxidation of polyunsaturated membrane fatty acids. 4-HNE accumulates under oxidative stress, contributes to cellular damage through covalent protein modification, and has also been reported to be increased in skin exposed to environmental pollutants [5,15,26,27,28]. As shown in Figure 3B, VL exposure significantly increased 4-HNE protein adduct formation, with a more pronounced effect at day 7, indicating enhanced oxidative stress and lipid peroxidation in exposed tissues. Of note, topical treatment with AOX Mix, either alone or following VL exposure, effectively prevented 4-HNE accumulation, maintaining levels comparable to untreated control samples at both time points analyzed. Finally, the expression of Heme oxygenase-1 (HO-1), a key gene involved in the cellular antioxidant response [29], was evaluated. Our results in Figure 3C demonstrated that VL exposure significantly upregulated HO-1 expression at day 7, consistent with the activation of oxidative stress-responsive pathways. In contrast, AOX Mix pre-treatment significantly attenuated this VL-induced increase, whereas no significant changes in HO-1 transcript levels were observed at day 2.

### 3.3. Protective Role of AOX Mix in VL-Induced Extracellular Matrix Degradation

**Figure 4:**
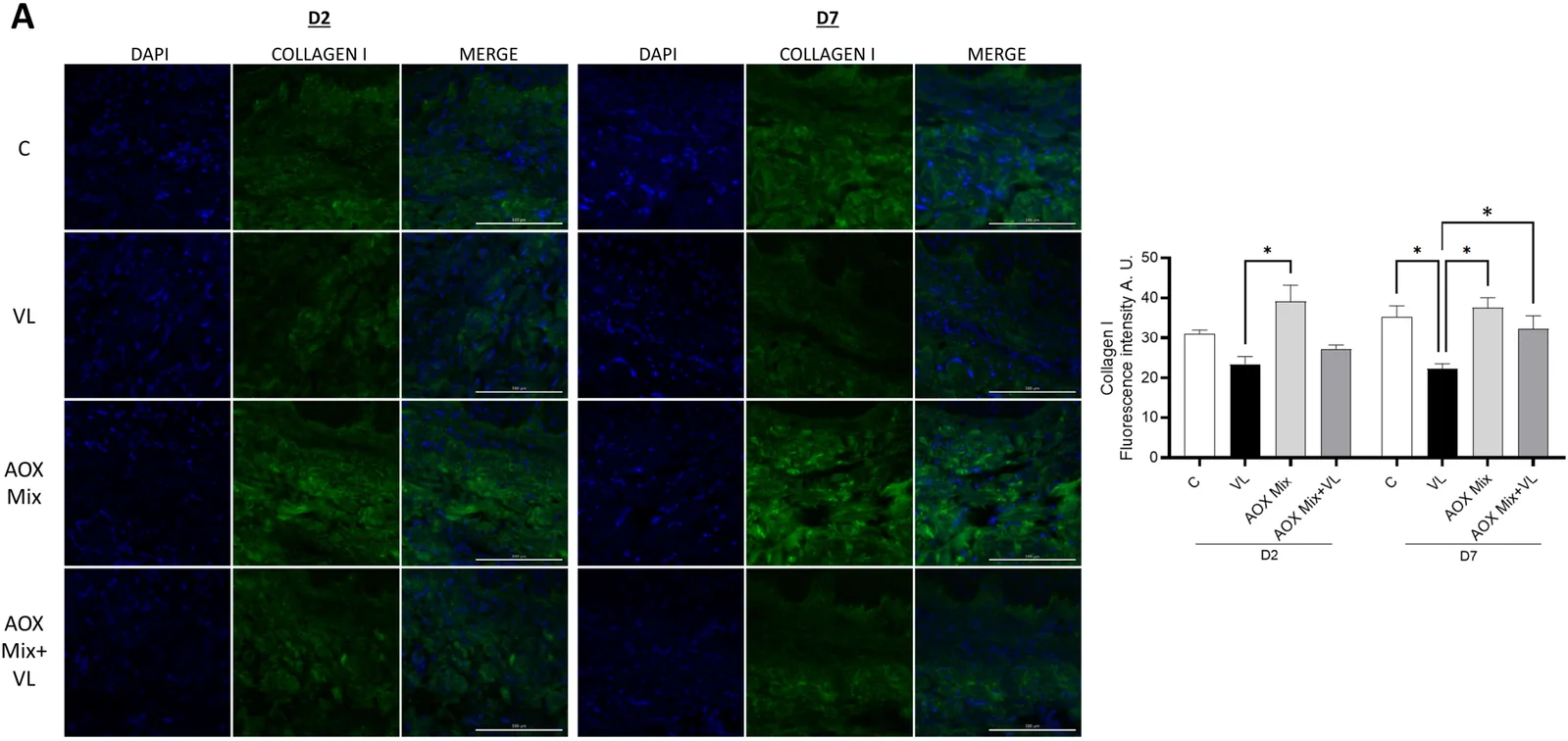

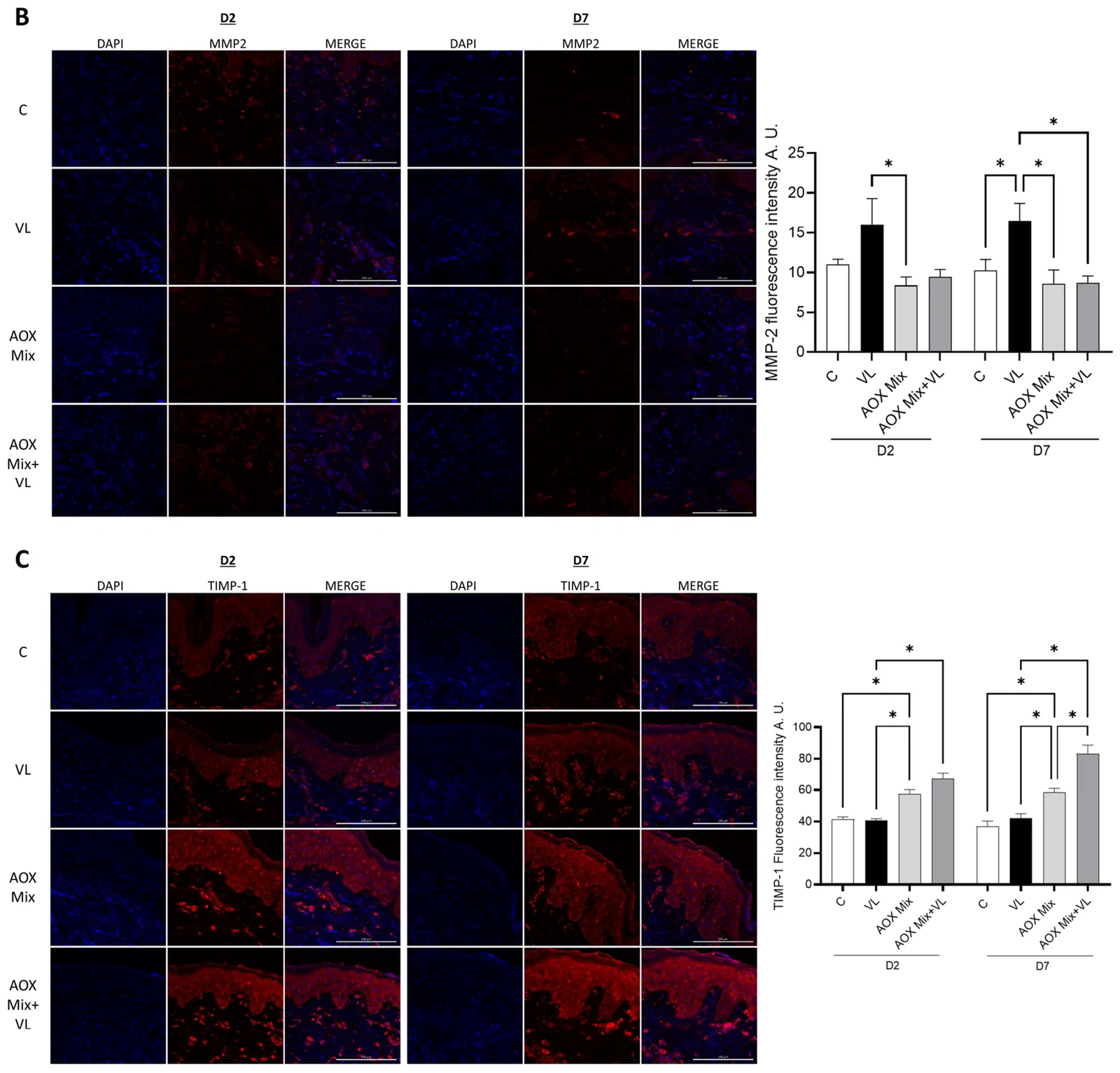
(**A**) Representative images of collagen I protein expression in ex vivo human skin biopsies after topical pre-treatment with AOX Mix formulation and exposure to VL radiation for 2 or 7 days (collagen-1, green; DAPI, blue). The right panel shows signal quantification of the fluorescence intensity. (**B**) Representative images of MMP2 protein expression in ex vivo human skin biopsies after topical pre-treatment with AOX Mix formulation and exposure to VL radiation for 2 or 7 days (MMP2, red; DAPI, blue). The right panel shows signal quantification of the fluorescence intensity. (**C**) Representative images of TIMP1 protein expression in ex vivo human skin biopsies after topical pre-treatment with AOX Mix formulation and exposure to VL radiation for 2 or 7 days (TIMP1, red; DAPI, blue). The right panel shows fluorescence intensity quantification. Images were captured at 40x magnification. Experimental groups: C, untreated control; VL, visible light exposure; AOX Mix, AOX Mix treatment alone; AOX Mix + VL, AOX Mix pre-treatment followed by visible light exposure. D2 and D7 indicate tissue collection on days 2 and 7, respectively. Significant differences are indicated by * *p*-value < 0.05. Data are expressed as mean ± SEM and analyzed using one-way ANOVA with Tukey’s multiple comparison post-hoc test.

Next, collagen type I was evaluated as a marker of extracellular matrix integrity, as it is the principal structural protein that maintains skin strength, resilience, and overall tissue architecture [30,31,32]. As shown in Figure 4A, VL exposure induced a decrease in collagen I protein expression, which was more pronounced on day 7, whereas pre-treatment with AOX Mix successfully prevented the loss induced by VL exposure. Moreover, the expression of MMP-2, an enzyme belonging to the matrix metalloproteinase (MMP) family and that is essential for maintaining tissue structure [33], was evaluated. As shown in Figure 4B, VL exposure significantly upregulated MMP-2 expression at day 7. Interestingly, this increase was successfully counteracted by AOX Mix pre-treatment at both days 2 and 7. In parallel, the expression of TIMP-1, a specific inhibitor of MMP that binds to metalloproteinase and prevents extracellular matrix degradation [34], was also evaluated. As depicted in Figure 4C, AOX Mix treatment significantly increased TIMP-1 expression at both days 2 and 7, either alone or in combination with VL exposure. Conversely, VL exposure alone did not induce significant changes in TIMP-1 expression. Collectively, these findings support the hypothesis that AOX Mix exerts a protective effect against VL-induced extracellular matrix remodeling by preserving collagen I expression, limiting MMP-2 upregulation, and enhancing TIMP-1 expression, thereby contributing to the preservation of extracellular matrix integrity.

### 3.4. AOX Mix Pre-Treatment Prevents 4HNE and Collagen Co-Localization Induced by VL Exposure

**Figure 5:**
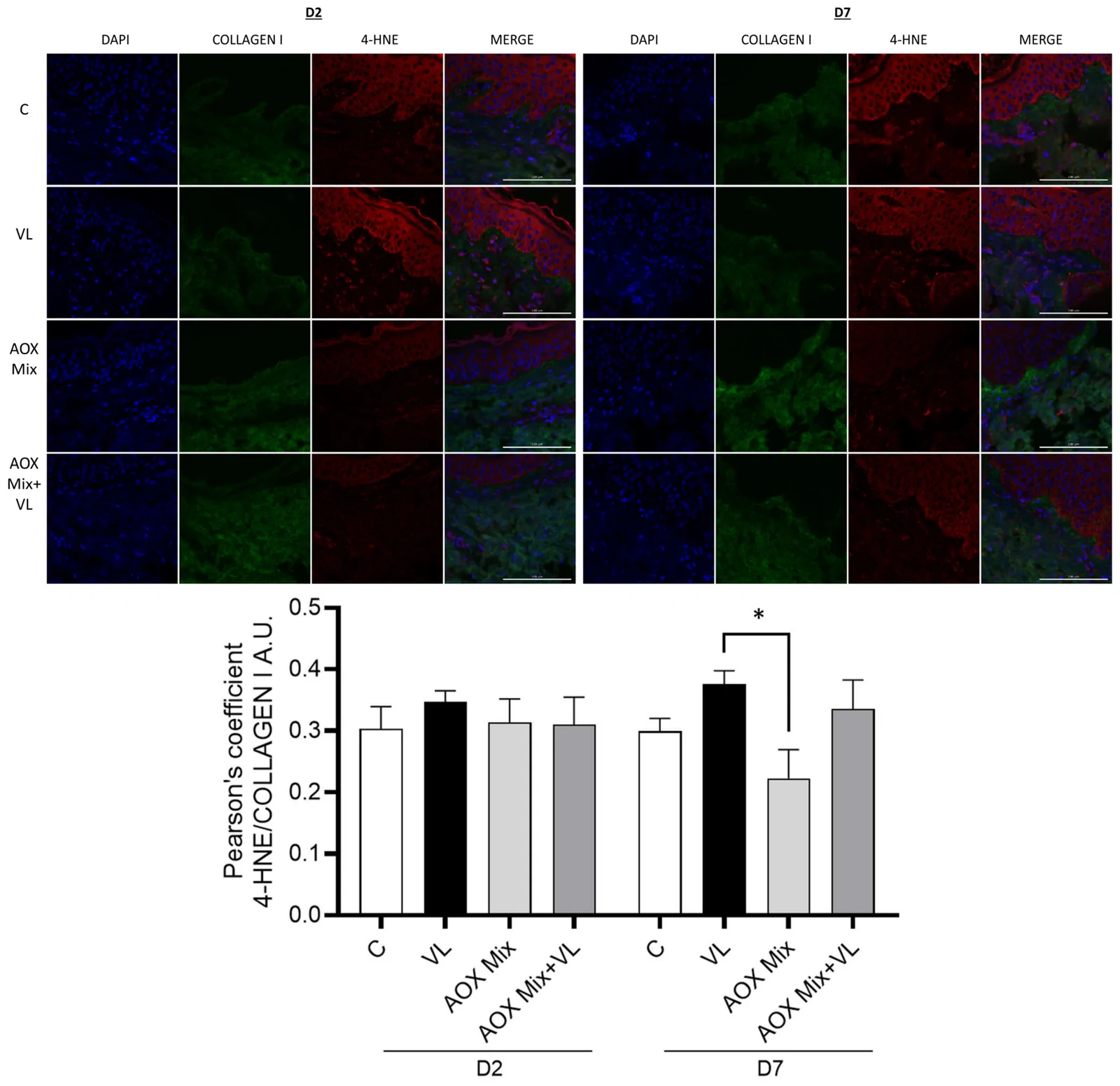
Representative images of co-immunofluorescence of 4HNE and collagen-1 protein expression in ex vivo human skin biopsies after topical pre-treatment with AOX Mix formulation and exposure to VL radiation for 2 or 7 days (4HNE, red; collagen-1, green; DAPI, blue). The lower panel shows signal quantification of Pearson’s Correlation Coefficient. Images were captured at 40x magnification. Experimental groups: C, untreated control; VL, visible light exposure; AOX Mix, AOX Mix treatment alone; AOX Mix + VL, AOX Mix pre-treatment followed by visible light exposure. D2 and D7 indicate tissue collection on days 2 and 7, respectively. Significant differences are indicated by * *p*-value < 0.05. Data are expressed as mean ± SEM and analyzed using one-way ANOVA with Tukey’s multiple comparison post-hoc test.

To further investigate whether the increased formation of 4-HNE protein adducts could contribute to the loss of collagen I expression, the co-localization of 4-HNE and collagen I was assessed using Pearson’s correlation coefficient, which quantifies the degree of co-localization between two fluorescent signals (ranging from −1 to +1, where values closer to +1 indicate stronger co-localization). Increased co-localization between 4-HNE and collagen I may indicate the formation of covalent 4-HNE adducts on collagen fibers, potentially leading to structural alterations of the extracellular matrix and partially contributing to the reduced collagen I expression. As shown in Figure 5, VL exposure increased 4-HNE/collagen I co-localization at day 7, whereas pre-treatment with AOX Mix significantly reduced this effect.

## 4. Discussion

Due to the widespread emphasis on UV radiation, the potential impact of VL is often underestimated in both clinical practice and public awareness. However, VL accounts for a substantial proportion of solar radiation reaching the Earth’s surface and is also emitted by artificial sources. Accumulating evidence indicates that VL can induce a range of biological effects in the skin, particularly photoaging, and therefore should be considered when evaluating environmental factors affecting skin health [35].

Interestingly, distinct wavelengths within the VL spectrum led to different biological responses, providing further insight into the mechanisms by which VL induces pigmentation. Indeed, it has been demonstrated that blue–violet light induces a stronger and more persistent pigmentation response than UVB exposure, especially in individuals with skin phototypes III and IV. In contrast, red light (630 nm) does not appear to induce hyperpigmentation, even at doses as high as 150 J/cm^2^ [36,37]. Furthermore, it is becoming clear that the blue–violet component of VL (415–455 nm) is the main contributor to long-lasting hyperpigmentation, as epidermal melanocytes can detect blue light through opsin 3 photoreceptors, which trigger melanin synthesis via tyrosinase activation [36,38,39]. Clinical studies have demonstrated that VL exposure, especially in combination with UVA radiation, can induce more intense and longer-lasting pigmentation in darker skin compared with lighter phototypes [40,41], highlighting VL as an important environmental factor contributing to pigmentary disorders. Although several studies have investigated the efficacy of topical formulations in reducing visible light-induced pigmentation, particularly tinted sunscreens enriched with iron oxides and pigmented titanium dioxide [42,43], the development of effective photoprotective strategies remains an unmet need, particularly for individuals with darker skin phototypes who are more susceptible to persistent hyperpigmentation. Collectively, these findings indicate that effective photoprotection should extend beyond UV radiation alone, and recognizing the contribution of VL to skin damage is therefore essential for developing more comprehensive preventive and therapeutic strategies [6].

For these reasons, the present study employed the TenSkin™ ex vivo skin model using biopsies from donors with dark skin phototypes, providing a physiologically relevant platform for investigating the molecular and histological effects of VL exposure and the protective efficacy of topical antioxidant formulations. This model enabled the evaluation of tissue-level responses within a physiologically relevant human skin environment, thereby enhancing the translational relevance of the present findings. Moreover, previous work from our laboratory demonstrated that maintaining physiological skin tension using the TenSkin TM model influences the timing and magnitude of skin responses to ozone exposure [14]. Together with the current findings, these observations highlight the importance of considering tissue tension when investigating skin responses to environmental stressors and evaluating potential photoprotective interventions.

As demonstrated by our data, VL exposure promoted melanin accumulation while preserving overall tissue morphology and viability. Indeed, histological analysis revealed no detectable structural alterations following VL exposure at either day 2 or day 7, indicating that the exposure was well tolerated and did not induce tissue damage. However, despite the absence of significant morphological abnormalities, VL exposure induced a marked increase in melanin deposition, particularly at day 7, as demonstrated by Fontana–Masson staining. This finding is consistent with previous studies showing that VL can stimulate melanogenesis and induce persistent pigmentation, especially after prolonged exposure, particularly in individuals with darker skin phototypes [44,45,46]. Moreover, the delayed increase in melanin accumulation observed at day 7 further supports the concept that VL could promote sustained pigmentary responses that may persist beyond the initial irradiation period, especially in individuals with darker skin phototypes, who possess larger, more highly melanized melanosomes and have a propensity for long-lasting pigmentation and dyschromia [47].

In addition, our data demonstrated increased accumulation of γH2AX and 4-HNE protein adducts, particularly after 7 days of VL exposure, suggesting the induction of DNA double-strand breaks, increased lipid peroxidation, and oxidative damage, as previously observed in other experimental models [48]. Moreover, Zastrow et al. [49] demonstrated that irradiation of ex vivo human skin biopsies with 400–700 nm VL induces oxidative damage, supporting our finding. Consistent with these observations, pre-treatment with the antioxidant formulation effectively prevented VL-induced melanin accumulation, γH2AX, and 4HNE accumulation, suggesting that oxidative stress plays a critical role in initiating VL-mediated melanogenesis. The combination of ascorbic acid, ferulic acid, and tocopherol, contained in the AOX Mix formulation, may act as a scavenger of reactive oxygen species generated during VL exposure, thereby limiting the activation of signaling pathways involved in melanin synthesis [50,51,52]. The ability of AOX Mix to attenuate melanin accumulation supports the concept that antioxidant-based interventions may represent a valuable complementary approach for preventing visible light-induced hyperpigmentation, particularly in individuals with darker skin phototypes.

In addition, the upregulation of HO-1 observed at day 7 provides further evidence for the activation of endogenous antioxidant defense mechanisms in response to VL-induced oxidative stress. HO-1 is a stress-inducible enzyme primarily regulated through activation of the Nrf2/Keap1 signaling pathway [53,54]. Under conditions of oxidative stress, ROS promote the dissociation of Nrf2 from Keap1, allowing its translocation to the nucleus, where it induces the expression of antioxidant response genes, including HO-1 [12,29]. HO-1 catalyzes the degradation of heme into biliverdin, carbon monoxide, and free iron [55]. Biliverdin is subsequently converted to bilirubin, both of which possess potent antioxidant properties, while carbon monoxide exerts anti-inflammatory and cytoprotective effects [55]. Therefore, increased HO-1 expression is generally considered an adaptive response that limits oxidative damage and restores redox homeostasis [56]. As a key enzyme that represents one of the first lines of antioxidant defense activated under conditions of increased oxidative stress [54], HO-1 expression was attenuated in AOX Mix-treated samples, suggesting that the formulation effectively reduced oxidative stress, thereby limiting the need to activate compensatory cellular defense pathways.

Visible light, especially in the blue–violet range, can promote dermal matrix remodeling mainly through oxidative stress. Skin chromophores such as flavins, porphyrins, opsins, melanin-related intermediates, and mitochondrial components can absorb VL and generate ROS. Consistent with previous studies and present findings, VL exposure increases oxidative damage and MMP expression in skin and reduces TIMP1 [11]. The increase in ROS can activate redox-sensitive signaling pathways, including AP-1 and NF-κB, which drive the expression of matrix-degrading enzymes such as MMP-1, MMP-3, and MMP-9. MMP-1 is especially relevant because it initiates the cleavage of type I collagen, while other MMPs further degrade collagen fragments and additional extracellular matrix components, ultimately promoting extracellular matrix degradation and structural weakening. Consistent with this mechanism, our results demonstrated a significant decrease in collagen I expression together with increased MMP-2 expression. In contrast, TIMP-1 expression was significantly increased following AOX Mix treatment, suggesting a shift toward preservation of extracellular matrix homeostasis. Collectively, these findings suggest that VL exposure may contribute to an imbalance between matrix metalloproteinases and their endogenous inhibitors [57] whereas AOX Mix partially counteracts these alterations.

Finally, our data demonstrated, for the first time, increased co-localization between collagen I and 4-HNE, suggesting the formation of covalent 4-HNE adducts on collagen fibers, which may contribute to the loss of collagen I following VL exposure. Indeed, 4-HNE has been shown to bind covalently to proteins, facilitating their ubiquitination and subsequent degradation through proteasomal pathways [58]. Overall, our findings provide further evidence that VL is an important contributor to cutaneous oxidative stress, pigmentation, and structural damage in darkly pigmented skin. The ability of AOX Mix to mitigate these responses underscores the potential of antioxidant-based interventions as a complementary strategy alongside conventional photoprotection and highlights the need to consider VL exposure when developing preventive and therapeutic approaches for photoaging and pigmentary disorders.

Although the present study provides novel insights into the effects of VL on skin, the underlying molecular mechanisms linking oxidative stress to extracellular matrix remodeling were not directly investigated. Future studies are needed to further define the pathways through which VL-induced oxidative stress contributes to collagen degradation and skin remodeling. In addition, although skin explants have inherent limitations, they remain one of the most physiologically relevant and reliable laboratory models for studying skin biology and treatment responses.

## Data Availability

All data of this study are included in this published article.

## Supplementary Materials

The following supporting information can be downloaded at: https://www.mdpi.com/article/10.3390/antiox15091072/s1, Figure S1: Emission spectrum of the visible-light LED array. Relative spectral intensity of the LED array used for visible-light exposure, measured at the sample position inside the incubator. The irradiance at the sample surface was 0.011 W/cm^2^, as measured using an MT-912 light meter.
